# A B Cell Epitope Peptide Derived from the Major Grass Pollen Allergen Phl p 1 Boosts Allergen-Specific Secondary Antibody Responses without Allergen-Specific T Cell Help

**DOI:** 10.4049/jimmunol.1501741

**Published:** 2017-01-16

**Authors:** Meena Narayanan, Raphaela Freidl, Margarete Focke-Tejkl, Ulrike Baranyi, Thomas Wekerle, Rudolf Valenta, Birgit Linhart

**Affiliations:** * Division of Immunopathology, Department of Pathophysiology and Allergy Research, Center for Pathophysiology, Infectiology, and Immunology, Medical University of Vienna, 1090 Vienna, Austria; and; † Section of Transplantation Immunology, Department of Surgery, Medical University of Vienna, 1090 Vienna, Austria

## Abstract

More than 40% of allergic patients suffer from grass pollen allergy. Phl p 1, the major timothy grass pollen allergen, belongs to the cross-reactive group 1 grass pollen allergens that are thought to initiate allergic sensitization to grass pollen. Repeated allergen encounter boosts allergen-specific IgE production and enhances clinical sensitivity in patients. To investigate immunological mechanisms underlying the boosting of allergen-specific secondary IgE Ab responses and the allergen epitopes involved, a murine model for Phl p 1 was established. A B cell epitope–derived peptide of Phl p 1 devoid of allergen-specific T cell epitopes, as recognized by BALB/c mice, was fused to an allergen-unrelated carrier in the form of a recombinant fusion protein and used for sensitization. This fusion protein allowed the induction of allergen-specific IgE Ab responses without allergen-specific T cell help. Allergen-specific Ab responses were subsequently boosted with molecules containing the B cell epitope–derived peptide without carrier or linked to other allergen-unrelated carriers. Oligomeric peptide bound to a carrier different from that which had been used for sensitization boosted allergen-specific secondary IgE responses without a detectable allergen-specific T cell response. Our results indicate that allergen-specific secondary IgE Ab responses can be boosted by repetitive B cell epitopes without allergen-specific T cell help by cross-linking of the B cell epitope receptor. This finding has important implications for the design of new allergy vaccines.

## Introduction

Grass pollen allergens are the most frequent elicitors of IgE-mediated allergies worldwide, provoking allergic symptoms such as rhinitis, conjunctivitis, allergic asthma, and atopic dermatitis (1–5). In Europe and the United States, >40% of allergic patients develop a clinically relevant sensitization to grass pollen (5). Group 1 allergens belong to the most important grass pollen allergens because they occur as highly cross-reactive allergens in pollen of most grass species, they are recognized by >90% of grass pollen–allergic patients, and they exhibit high allergenic activity as demonstrated in several studies (2, 6, 7). Phl p 1 from timothy grass pollen contains most of the group 1–specific IgE and T cell epitopes and therefore serves as a diagnostic marker for grass pollen allergy and is considered as a representative group 1 allergen for grass pollen allergen-specific immunotherapy (SIT) (8–12). A recent study that has investigated the development of grass pollen allergy in childhood even suggested that sensitization to Phl p 1 may be an initiating event in allergic sensitization to grass pollen (13). Once sensitization has occurred, repeated and subsequent exposure to grass pollen leads to increases of allergen-specific IgE production after the pollen season and thus may be responsible for progression of silent sensitization to allergic symptoms and, in already symptomatic patients, for progression to more severe symptoms (13, 14). Only SIT but not pharmacological therapy has been shown to prevent this progression toward more severe symptoms (i.e., allergic rhinitis to asthma) in children (15) and was found to have long-term effects even after discontinuation (16). Likewise, it was found that SIT reduced the boosts of IgE production (17–19) whereas suppression of T cell responses by systemic cyclosporine (20) and even loss of T cell function in patients suffering from AIDS (21) did not prevent allergen-induced boosts of IgE production in allergic patients. Likewise, costimulation blockade in a murine model of grass pollen allergy only suppressed IgE sensitization but did not have effects on secondary IgE responses (22), and costimulation blockage also did not ameliorate IgE-mediated asthma (23).

In this study, we investigated in a mouse model to which extent B cell and T cell epitopes derived from the major grass pollen allergen Phl p 1 are involved in boosting secondary IgE responses. We prepared a recombinant fusion protein consisting of a major IgE epitope–containing portion of Phl p 1 and of an allergen-unrelated viral carrier protein, the PreS surface protein from hepatitis B, for induction of IgE sensitization toward Phl p 1 (24). In this model T cell help for allergen-specific IgE induction comes from the allergen-unrelated carrier protein without involvement of allergen-specific T cells. We then studied the boosting of allergen-specific IgE responses with constructs containing the allergen-specific B cell epitope in a monomeric, dimeric, and oligomeric form without allergen-specific T cell epitopes. Our finding that oligomeric B cell epitopes can induce boosts of IgE production without detectable allergen-specific T cell responses has implications for the development of immunotherapy strategies seeking to control the boosts of secondary IgE production.

## Materials and Methods

### Synthesis of Phl p 1– and Bet v 1–derived peptides, MALDI-TOF

A 30-aa peptide derived from the major grass pollen allergen Phl p 1 ranging from aa 212 to 241 (VRYTTEGGTKTEAEDVIPEGWKADTSYESK) was synthesized using a Wang resin from AAPPTec (Louisville, KY) on a Liberty microwave peptide synthesizer from CEM (Darmstadt, Germany). Additionally, the same peptide was prepared containing an N-terminal cysteine residue as previously described (25). For control purposes, an irrelevant peptide derived from the unrelated major birch pollen allergen Bet v 1 (VDHTNFKYNYSVIEGGPIGDTLEKISNEIK) was synthesized. Peptides were purified via preparative HPLC (Thermo Fisher Dionex, Vienna, Austria) using a Jupiter 4-μm Proteo 90 Å, Axia packed column (Phenomenex, Aschaffenburg, Germany), and the mass was confirmed by MALDI-TOF mass spectrometry. For this purpose, purified synthetic peptides were dissolved in water and mixed with matrix solution (saturated solution of α-cyano-4-hydroxycinnamic acid in 50% acetonitrile, and 2.5% trifluoroacetic acid) in a 1:1 ratio and deposited onto the target and air-dried. Laser desorption mass spectra were acquired in a linear mode using a Bruker microflex mass spectrometer (Bruker, Billerica, MA).

### SDS-PAGE, gel filtration

Purified synthetic peptides were analyzed by means of SDS-PAGE under reducing and nonreducing conditions. Nonreducing conditions were obtained by dissolving the peptides in sample buffer without 2-ME. Samples were separated in 18% SDS-PAGE without boiling. Gels were subsequently stained with Coomassie blue (26).

For gel filtration, 300-μl aliquots of the peptides (monomeric peptide [1xP], concentration [*c*] = 1 μg/μl; dimeric peptide [2xP], *c* = 1 μg/μl) were loaded onto a Superdex peptide 10/300 GL column (GE Healthcare, Uppsala, Sweden) at room temperature and equilibrated with 10 mM phosphate buffer (pH 7.4) containing 137 mM NaCl. The flow rate was 0.5 ml/min, fractions of 1 ml were collected, and the absorbance was measured at 280 nm. Fractions showing a peak were further analyzed by MALDI-TOF. Dextran blue (2000 kDa), a peptide dimer (7.8 kDa), and a peptide monomer (3.9 kDa) were used as standards.

### Preparation of immunogens, rabbit antisera

**FIGURE 1.**
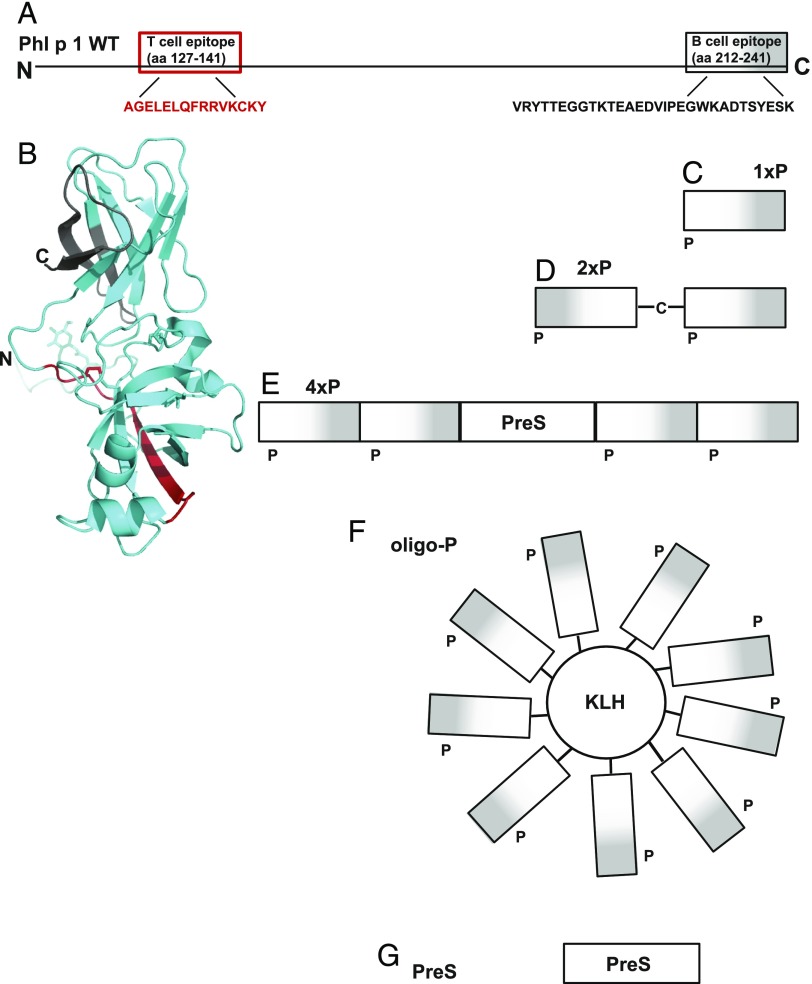
Schematic representation of the molecules used for sensitization and boosting. (**A**) Schematic and (**B**) structural ribbon representation of the Phl p 1 allergen. Indicated are the N and C termini, the T cell epitope (red), and the B cell epitope (white to gray with increasing darkness from the N to C terminus). Molecules used for sensitization and/or boosting are (**C**) the isolated peptide (1xP), (**D**) a dimeric peptide (2xP) obtained by cross-linking via N-terminal cysteines of peptide P, (**E**) a recombinant fusion protein containing two peptides at the N terminus and two at the C terminus of hepatitis B–derived PreS (4xP), (**F**) KLH containing several copies of the peptide P attached by chemical coupling (oligo-P), and (**G**) recombinant PreS alone.

The Phl p 1–derived peptide (peptide P) was chosen for this study owing to the lack of Phl p 1–specific T cell epitopes recognized by BALB/c mice (Fig. 1) (27). Molecules containing different numbers of peptide P were prepared (Fig. 1). The synthetic monomeric peptide without cysteine (1xP) was dissolved in water and aliquots were stored at −20°C. A peptide dimer (2xP) was prepared from the cysteine-containing synthetic peptide by cross-linking as previously described (28). A molecule containing four copies of peptide P (4xP) was produced as a recombinant fusion protein that contained hepatitis B–derived PreS as a carrier and two copies of P at the N terminus as well as at the C terminus (24). To prepare a molecule containing several copies of peptide P (oligomeric P [oligo-P]), the synthetic peptide was coupled to maleimide-activated KLH (Sigma-Aldrich, St. Louis, MO) via an N-terminal cysteine residue according to the manufacturer’s instruction. An oligomeric immunogen containing another carrier (referred to as peptide–OVA conjugate) was prepared by coupling peptide P to maleimide-activated OVA via cysteine residues following the manufacturer’s instructions (Pierce, Rockford, IL). Rabbit antisera specific for 1xP, 2xP, 4xP, oligo-P, PreS, and KLH were obtained by immunization of rabbits with the Ags in CFA and incomplete CFA (Charles River Laboratories, Kisslegg, Germany) as described (25).

### Circular dichroism measurement

Circular dichroism (CD) measurements of purified peptides 1xP and 2xP dissolved in water were performed on a Jasco J-810 spectropolarimeter (Jasco, Tokyo, Japan) using a 1-mm quartz cuvette. Spectra were recorded at a scan speed of 50 nm/min at room temperature and were corrected by subtracting the corresponding baseline spectrum, which was measured for water at identical conditions. Results are means of three measurements and are expressed as molecular ellipticity (29).

### Dot blots

The proteins 1xP, 2xP, 4xP, and oligo-P, each representing 1 μg of peptide (P) [i.e., 1 μg of 1xP (*c* = 1 μg/μl in water), 1 μg of 2xP (*c* = 1 μg/μl in water), 2.2 μg of 4xP (*c* = 0.7 μg/μl in 10 mM Tris, 200 mM Nacl [pH 7.5]), 1 μg of oligo-P, calculated according to the coupling efficiency (*c* = 1 μg/μl in PBS)], PreS and KLH in equimolar amounts as contained in 4xP and oligo-P [i.e., 1.81 μg of PreS (*c* = 0.1 μg/μl in 10 mM NaH_2_PO_4_ [pH 4.8]), 1 μg of KLH, calculated according to the coupling efficiency (*c* = 5 μg/μl in water)], and 1 μg of an irrelevant Ag (the unrelated major birch pollen allergen Bet v 1, *c* = 1 μg/μl in water) for control purposes were dotted onto nitrocellulose membrane strips (Schleicher & Schuell BioScience, Dassel, Germany), air-dried, and blocked with 3% nonfat dry milk powder (Bio-Rad Laboratories, Hercules, CA) for 2 h at room temperature. The strips were then incubated at 4°C overnight with respective rabbit antisera (i.e., anti–oligo-P, anti-4xP, anti-PreS, anti-KLH, and rabbit preimmune serum) diluted 1:10,000. Bound Abs were then detected with 1:5000 diluted [^125^I]-labeled goat anti-rabbit IgG Ab (PerkinElmer, Waltham, MA) and visualized by autoradiography.

### Immunization and boosting of mice

Female 6- to 8-wk-old BALB/c mice were purchased from Charles River Laboratories (Sulzfeld, Germany) and kept in the animal care unit of the Department of Pathophysiology and Allergy Research, Medical University of Vienna (Vienna, Austria) according to the local guidelines of laboratory animal care. All animal experiments were approved by the local Ethics Committee.

In a first pilot experiment, we studied whether IgE Abs and an allergic immune response could be induced against peptide P by sensitization with 4xP. Eight mice were sensitized with an s.c. injection of 44 μg of 4xP (i.e., 20 μg of P) adsorbed to Al(OH)_3_ (Alu-Gel-S; SERVA Electrophoresis, Heidelberg, Germany) on day 1. Blood samples were collected from tail veins of the mice on day 0 (preimmune serum) and on day 22 after sensitization (immune serum) to investigate the induction of peptide P–specific IgE responses. A group of mice (*n* = 5) was sensitized and then boosted three times by s.c. injections of Al(OH)_3_-adsorbed 4xP (44 μg) in 4-wk intervals. On day 180, spleens were removed under aseptic conditions for preparation of splenocytes and analysis of T cell responses by violet proliferation dye (VPD)450 FACS experiments and thymidine incorporation assays. Sera from these mice obtained on day 180 were later used to sensitize rat basophilic leukemia (RBL) cells for testing the molecules used for boosting.

In a second set of experiments we studied whether sensitization with peptide P (i.e., 2xP) without carrier will induce peptide P–specific Ab responses. For this purpose, an additional five mice were sensitized on day 1 with an s.c. injection of Al(OH)_3_-adsorbed 2xP (20 μg of P, i.e., an equimolar amount of P as compared with 4×P used in the first pilot experiments) and boosted on day 165 with an s.c. injection of 2xP (20 μg of P) in PBS without adjuvant. Peptide-specific IgE and IgG Ab responses were analyzed in blood samples obtained on days 0, 7, 14, 28, 42, 164, 172, and 179 by ELISA.

**FIGURE 2.**
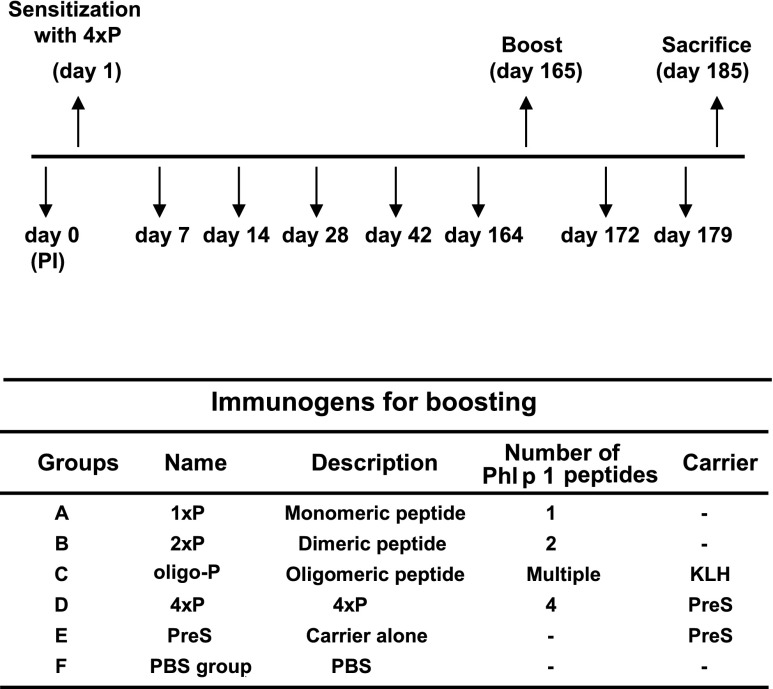
Mouse model for primary and secondary allergen-specific IgE responses. According to the scheme (upper part) groups of BALB/c mice (A–F, *n* = 5) were sensitized to 4xP on day 0, blood samples were taken as indicated, and a single boost was given on day 165 with different molecules or PBS (groups A–F) as shown below. In the upper part, sensitization, boost, and sacrifice are indicated by an upward-pointing arrow and the bleedings by a downward-pointing arrow.

In a third set of experiments, we studied whether IgE and IgG responses induced by sensitization with 4xP can be boosted with different immunogens. Fig. 2 provides an overview of these experiments. Five groups of BALB/c mice (groups A–E, *n* = 5) were sensitized s.c. on day 1 with 44 μg of 4xP (i.e., 20 μg of P) adsorbed to Al(OH)_3_. A control group (i.e., group F) received only PBS and Al(OH)_3_ (Fig. 2). Blood samples were taken on days 0, 7, 14, 28, 42, and 164 to monitor IgE and IgG Ab responses. On day 165, when IgE responses had declined, mice were s.c. boosted with different immunogens dissolved in PBS or PBS alone (i.e., group A, 1xP; group B, 2xP; group C, oligo-P; group D, 4xP; group E, PreS; group F, PBS). Immunogens containing peptide P were adjusted to contain equimolar amounts (i.e., 20 μg) of peptide P, and equimolar amounts (i.e., 20 μg) of carrier (PreS) as contained in 4xP were used in the booster injections. Blood samples were collected from tail veins and the sera were stored at −20°C until analysis. Results from each of the three sets of experiments were obtained from two independent mouse immunizations. The third set of experiments was repeated in larger mouse groups (*n* = 8) and results were added in Supplemental Figs. 1–4.

### FACS analysis

Single-cell suspensions were prepared from isolated mouse spleens (*n* = 5), labeled with VPD450 (BD Biosciences, San Diego, CA) according to the manufacturer’s instructions, and cultivated for 4 d in the presence of peptide 1xP (0.3 μg/well), the carrier molecule PreS (1 μg/well), Con A (0.5 μg/well), or medium. Subsequently, cells were stained with FITC-labeled rat anti-mouse CD4 Ab (clone RM4-5, isotype: rat IgG2a, k; BD Biosciences) or isotype control (FITC rat IgG2a, k clone R35-95; BD Biosciences). Cells were analyzed on a FACSCanto (BD Biosciences, Franklin Lakes, NJ). Data of flow cytometry were analyzed in FlowJo version 10 (Miltenyi Biotec). Lymphocytes were gated in forward and side scatter to remove cell debris. Lymphocytes were further gated for CD4^+^ cells and analyzed for proliferation rates of CD4^+^ and dilution of VPD450 after stimulation as indicated. Representative blots of stimulated splenocytes of one mouse are demonstrated (30). FACS results were obtained from two independent experiments.

### ELISA experiments

Peptide-specific Ab responses in mice were analyzed by ELISA as described. ELISA plates (Nunc MaxiSorp, Roskilde, Denmark) were coated with 2xP (5 μg/ml), blocked with 1% (w/v) BSA in PBST for 2 h, and incubated with mouse sera diluted 1:10 for IgE and 1:500 for IgG_1_ measurements. Bound IgE Abs were detected with an HRP-conjugated goat anti-mouse IgE antiserum (SouthernBiotech, Birmingham, AL) diluted 1:6000. Bound IgG Abs were detected with rat anti-mouse IgG_1_ mAb (BD Pharmingen, San Diego, CA) diluted 1:1000, followed by an HRP-conjugated goat anti-rat antiserum (Amersham Biosciences, Buckinghamshire, U.K.) diluted 1:2000. The specificity of the goat anti-mouse IgE antiserum for mouse IgE was confirmed by ELISA, as it detected mouse monoclonal IgE (28) but not mouse monoclonal IgG_1_ Abs (31) (data not shown). All values were calculated by subtraction of the appropriate buffer controls (22, 32).

### RBL assay

RBL cell mediator release assays were carried out as described (28). RBL-2H3 cells were cultivated in 96-well tissue culture plates (6 × 10^5^ cells per well) for 20 h at 37°C using 5% CO_2_.

Sensitization of 4xP-immunized mice (*n* = 8) was tested by loading individual sera (1:10 diluted) for 2 h on RBL cells and by induction of mediator release adding 0.3 μg/ml peptide–OVA conjugate.

To test the ability of the different immunogens (1xP, 2xP, oligo-P, 4xP, PreS, KLH) to cross-link peptide P–specific IgE Abs on basophils, RBL cells were loaded with 1:10 diluted pooled sera obtained on day 180 from mice sensitized and boosted three times with 4xP. Cells were then washed twice with Tyrode’s buffer (137 mM NaCl, 2.7 mM KCl, 0.5 mM MgCl_2_, 1.8 mM CaCl_2_, 0.4 mM NaH_2_PO_4_, 5.6 mM d-glucose, 12 mM NaHCO_3_, 10 mM HEPES, and 0.1% [w/v] BSA [pH 7.2]) to remove unbound Abs. Cells were exposed to each immunogen containing 0.3 μg/ml peptide P. β-Hexosaminidase release was analyzed after 1 h.

To measure peptide-specific release in mice from groups A–F, sera from each mouse group were pooled due to serum limitations and loaded on RBL cells in 1:20 dilutions for 2 h. Degranulation of RBL cells was induced by adding 0.3 μg/ml peptide–OVA conjugate. All results were expressed as percentages of total β-hexosaminidase released after the addition of 1% Triton X-100 and were obtained from two independent experiments. The values representing the spontaneous release were subtracted and all values represent the mean of triplicate determinations.

### Proliferation of splenocytes

Spleens were removed under aseptic conditions (day 185) and homogenized. After lysis of erythrocytes, cells were washed and resuspended in supplemented medium (RPMI 1640, 10% FCS, 2 mM glutamine, and 0.1 mg/ml gentamicin). Single-cell suspensions were plated into 96-well round-bottom plates at a concentration of 2 × 10^5^ cells per well in triplicates and stimulated with either peptide 1xP (0.3 μg/well), irrelevant peptide (0.3 μg/well), PreS (2 μg/well), or Con A (0.5 μg/well) as positive control. The cultures were then pulsed with 0.5 μCi/well tritiated thymidine for 16 h and harvested. The proliferation responses were measured by scintillation counting on a Wallac MicroBeta TriLux scintillation counter (PerkinElmer). The ratio of the mean proliferation after Ag stimulation and medium control values, that is, the stimulation index (SI), was calculated.

### Statistical analysis

Comparison of Ab levels within each mouse group was performed by the Wilcoxon signed rank test for paired data using SPSS software. A *p* value < 0.05 was considered statistically significant. Results are either shown as box plots with indicated medians using SPSS software version 20.0 or column bar graphs using GraphPad Prism (GraphPad Software) version 5.0.

## Results

### Characterization of proteins and peptides for the dissection of IgE and T cell reactivity in murine models of primary and secondary allergic immune responses

To investigate the contribution of B and T cell epitopes on secondary allergen-specific IgE Ab responses, we followed the hapten-carrier principle established by Benacerraf and constructed a fusion protein consisting of a peptide from the IgE epitope–containing region of the major timothy grass pollen allergen, Phl p 1, and an unrelated carrier protein (i.e., PreS from hepatitis B virus) providing T cell help (Fig. 1) (24).

The Phl p 1 peptide P comprised 30 aa of the C terminus of Phl p 1 (peptide P, aa 212–241: VRYTTEGGTKTEAEDVIPEGWKADTSYESK) where the major IgE epitopes of allergic patients are located (25) (Fig. 1A, 1B, gray). Carrier-bound peptide P has been shown to induce strong Phl p 1–specific Ab responses, inhibiting grass pollen allergic patients’ IgE binding to Phl p 1 (25). Importantly, peptide P does not contain the Phl p 1 T cell epitope recognized by BALB/c mice (27) (Fig. 1A, 1B, red). Thus, peptide P contains exclusively B cell epitopes.

**FIGURE 3.**
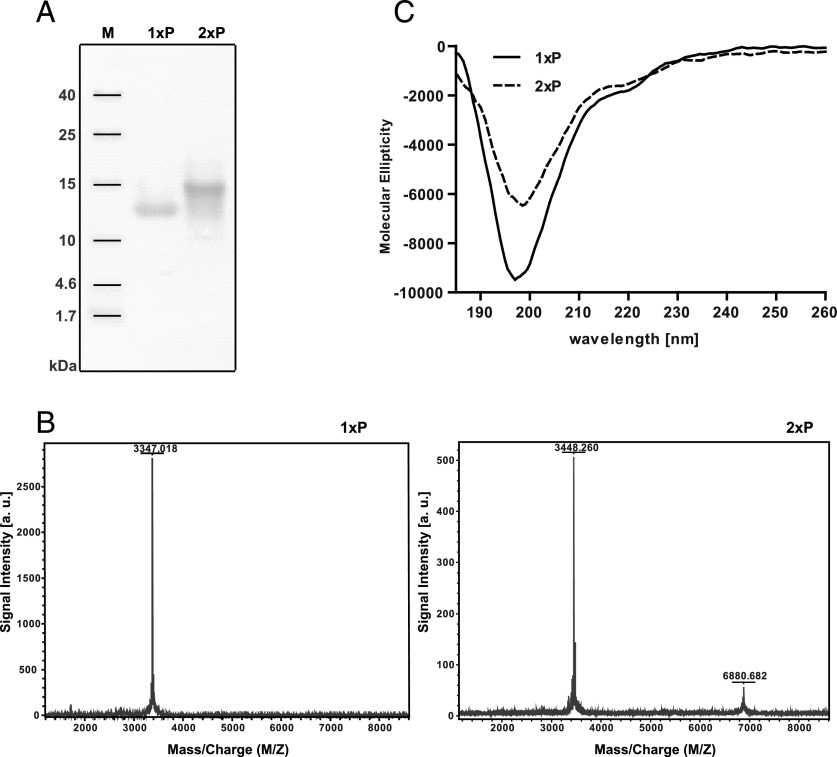
Biochemical and biophysical characterization of the Phl p 1–derived peptide. (**A**) Coomassie blue–stained SDS-PAGE containing monomeric peptide (lane 1xP), dimeric peptide (lane 2xP), and a molecular mass marker (lane M) under nonreducing conditions. Molecular masses are displayed in kiloDaltons at the left margin. (**B**) Mass spectrometric analysis of the monomeric (1xP) and dimeric peptide (2xP). The mass/charge ratios (*x*-axes, M/Z) and the signal intensities (*y*-axes, arbitrary units) are shown. (**C**) Far UV–CD spectra of the monomeric (1xP) and dimeric (2xP) peptide. The molecular ellipticities (*y*-axis) at different wavelengths (*x*-axis) are displayed.

Pure monomeric peptide P (i.e., 1xP) was obtained by chemical synthesis, and its experimental molecular mass determined by mass spectrometry (i.e., 3347.018 Da) (Figs. 1C, 2, 3A, 3B, left panel) closely corresponded to the mass calculated according to the sequence (i.e., 3348.58 Da). According to CD analysis, 1xP showed random coil conformation (Fig. 3C) and was monomeric under physiological conditions as demonstrated by gel filtration experiments (data not shown). A dimeric form of peptide P (2xP) was obtained by addition of an N-terminal cysteine residue to the peptide sequence, which resulted in the formation of disulfide bonds (Figs. 1D, 3A). In fact, the 2xP preparation contained a monomeric and dimeric form as revealed by SDS-PAGE (Fig. 3A), mass spectrometry (Fig. 3B, right panel), and gel filtration (data not shown). According to SDS-PAGE and gel filtration, ∼30% of the peptide preparation was monomeric. Circular dichroism analysis revealed that 2xP assumed a similar random coil conformation as 1×P (Fig. 3C).

**FIGURE 4.**
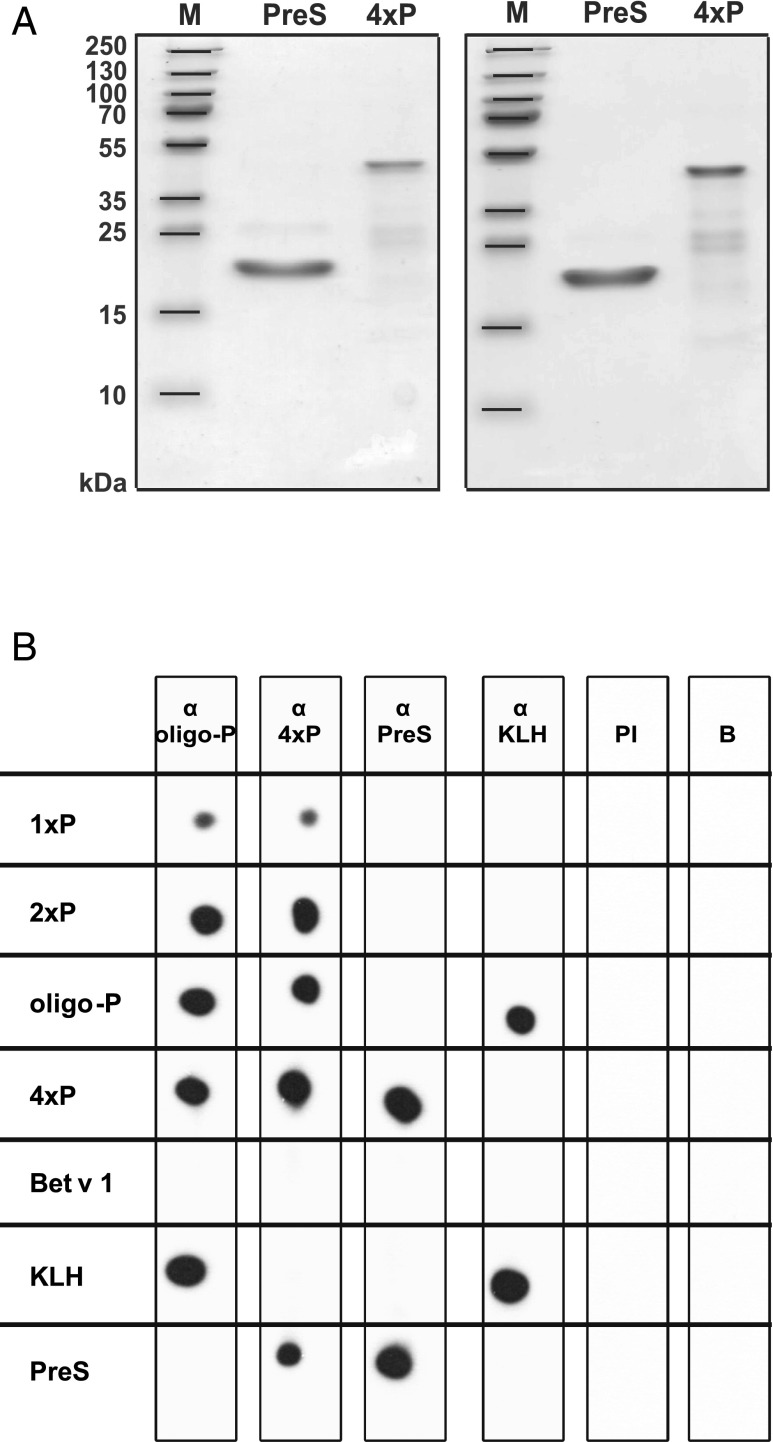
(**A**) Coomassie-stained SDS-PAGE containing purified PreS and 4xP under reducing (left panel) and nonreducing conditions (right panel). Lane M represents the molecular mass marker. (**B**) Immunological characterization of the immunogens. Nitrocellulose-dotted immunogens (1xP, 2xP, oligo-P, 4xP, Bet v 1, KLH, and PreS) were probed with specific Ab probes (lanes α oligo-P, α 4xP, α PreS, α KLH), normal rabbit serum (lane preimmune sera [PI]), or buffer alone (lane B). Bound Abs were detected with [^125^I]-labeled goat anti-rabbit IgG and visualized by autoradiography.

Furthermore, two constructs were made consisting of peptide P and T cell epitope–containing carrier proteins. The fusion protein 4xP contained four copies of peptide P: two attached to the N terminus and two attached to the C terminus of the hepatitis B virus–derived surface protein PreS (Fig. 1E) as well as PreS alone (Fig. 1G) were expressed in *Escherichia coli* and purified (Fig. 4A). Both PreS and 4xP appeared as monomeric proteins according to their migration behavior under reducing and nonreducing conditions in SDS-PAGE (Fig. 4A).

In an additional construct, multiple copies of peptide P were bound to another heterologous carrier, that is, the carrier molecule KLH, to yield oligo-P (Fig. 1F).

We then tested whether peptide P is accessible to Ab binding in the peptides (1xP, 2×P), in the recombinant fusion protein (4xP), and in the KLH conjugate (oligo-P) in their native form by dot blotting (Fig. 4B). Dot-blotted peptides and proteins were exposed to rabbit antisera specific for oligo-P, 4xP, PreS, and KLH. Anti–oligo-P Abs reacted with each of the components containing the peptide P (i.e., 1xP, 2xP, 4xP, oligo-P). They also recognized KLH, as oligo-P represented a peptide P–KLH conjugate (Fig. 4B, lane α oligo-P). Anti-4xP antisera reacted with the peptide-containing molecules and the carrier PreS (lane α 4xP). PreS could be detected by PreS-specific Abs in 4xP and PreS (lane α PreS), and KLH-specific antisera bound to oligo-P and KLH (lane α KLH). No reaction was found with the rabbits’ preimmune antisera or buffer (lanes PI and B). The unrelated control allergen Bet v 1 showed also no reactivity (Fig. 4B).

### Induction of peptide P–specific IgE responses with carrier-specific T cell help in BALB/c mice

**FIGURE 5.**
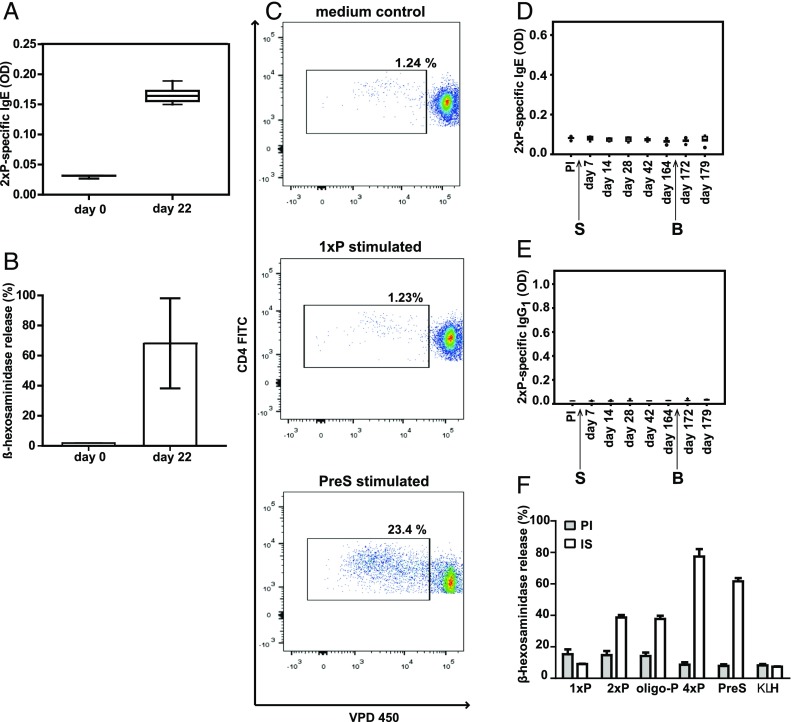
Demonstration of the carrier specificity of the T cell response and the induction of peptide-specific IgE. (**A**) Peptide-specific IgE Ab levels of BALB/c mice (*n* = 8) before immunization (day 0) and after sensitization (day 22) with 4xP (*x*-axis). Shown are IgE levels specific for 2xP (*y*-axis, OD values as box plots with indicated medians). (**B**) RBL cells were loaded with serum IgE obtained before immunization (day 0) and after sensitization of mice (*n* = 8) with 4xP (day 22). Displayed are the mean β-hexosaminidase releases ± SD (*y*-axis, percentages of total release) after exposure to an OVA–peptide conjugate containing several copies of peptide P. (**C**) Proliferation of VPD450-labeled splenocyte cells from one representative mouse sensitized and boosted three times with 4xP after in vitro stimulation with 1xP, PreS, or medium. Gates indicate percentages of CD4^+^ proliferating T cells of total CD4^+^ T cells. Lack of induction of peptide-specific IgE (**D**) and IgG_1_ (**E**) Ab responses in mice sensitized and boosted once only with 2xP. Shown are Ab levels specific for 2xP (*y*-axes, OD values as box plots with indicated medians) measured before and at different days after sensitization (S) and boosting (B) (*x*-axes). (**F**) Induction of degranulation of RBL cells loaded with serum IgE (day 180) obtained from mice (*n* = 4) immunized with 4xP. Cells loaded with preimmune sera (PI; filled columns) or immune sera (IS; open columns) were exposed to different molecules (*x*-axis, 1xP, 2xP, oligo-P, 4xP, PreS, KLH). The mean β-hexosaminidase releases ± SD are shown as percentages of the total releases on the *y*-axis.

In a first set of experiments we investigated whether the PreS fusion protein 4xP, which contains four copies of the B cell epitope–containing peptide P, can be used to induce a peptide P–specific IgE response in BALB/c mice. Fig. 5A and 5B show that a peptide-specific IgE Ab response could be induced with a single sensitization and that the peptide-specific IgE Abs can be used to sensitize RBL cells, which then can be degranulated with a peptide–OVA conjugate containing several copies of the peptide (Fig. 5B). We then analyzed the specificity of the T cell responses in the 4xP-sensitized mice. To enhance the T cell response, three booster injections with 4xP were given. Splenocytes isolated from 4xP-immunized and -boosted mice were labeled with VPD and stimulated with the peptide (1xP), carrier (PreS), or Con A. Proliferation of CD4^+^ T cells could only be detected in response to the carrier (mean, 15.62% of total CD4^+^ cells), but not in response to the peptide (mean, 1.89% of CD4^+^ cells), which was in the range of the medium control (mean, 1.72% of CD4^+^ cells). Con A–stimulated cells showed a mean proliferation of 68.56% (Fig. 5C, Supplemental Fig. 1). No difference in proliferation of CD4^+^ cells was detected between unstimulated and 1xP-stimulated cells, whereas proliferation in response to PreS was significantly increased (Supplemental Fig. 1). Similar results were obtained by thymidine incorporation assays. Mouse splenocytes were stimulated with peptide 1xP, PreS, Con A, or medium and [^3^H]thymidine uptake was measured (peptide P, SI = 1; PreS, SI = 5; Con A, SI = 18). These results show that immunization of mice with 4xP induced a peptide-specific IgE Ab response without detectable peptide-specific T cell help whereas carrier-specific T cells were detected.

The lack of T cell epitopes recognized by BALB/c mice in peptide P was also confirmed by a separate immunization experiment. Mice that were sensitized with Al(OH)_3_-adsorbed 2xP and boosted with 2xP did not develop any detectable peptide-specific IgE or IgG_1_ response (Fig. 5D, 5E).

### IgE Abs induced by immunization with 4xP and then bound to RBL cells are cross-linked by 2xP, oligo-P, 4xP, and PreS, but not by 1xP and KLH

Next we performed basophil activation experiments to determine whether 4xP-induced IgE Abs induce cross-linking of basophil-bound IgE and degranulation. For this purpose, RBL cells were loaded with serum IgE obtained from 4xP-sensitized and -boosted mice (Fig. 5A–C) on day 180 and exposed to Ags that were intended to be used in boost experiments. These Ags included peptide P in various forms (i.e., monomeric, 1xP; dimeric, 2xP; oligomeric, oligo-P), tetrameric peptide P fused to the carrier PreS (4xP), or Ags lacking peptide P (i.e., carriers PreS and KLH) (Fig. 5F). We found that only those components containing at least two copies of P (i.e., 2xP, oligo-P, 4xP) were able to cross-link receptor-bound IgE Abs and to induce a β-hexosaminidase release from RBL-2H3 cells (2xP, 38.6%; oligo-P, 37.7%). 1xP induced only 9% degranulation, which was in the range of the release when preimmune sera were loaded on the cells. This result confirmed that 1xP represented a monomer (Fig. 5F). 4xP induced the highest release (77.4%), as it cross-linked not only peptide P but also PreS-specific IgE Abs. The carrier KLH, which was not included in 4xP, did not induce a specific RBL degranulation (7.4%), whereas PreS alone induced degranulation (61.6%) due to the presence of PreS-specific IgE in sera from mice sensitized to 4xP (data not shown) (Fig. 5F). An average background degranulation of 11.5% was observed when RBL cells were loaded with the preimmune sera and challenged with the different immunogens.

### Peptide-specific IgE Ab responses are boosted significantly by oligo-P

**FIGURE 6.**
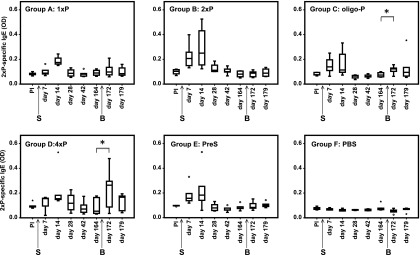
Peptide-specific IgE Ab levels over time in groups of mice (groups A–F). Shown are IgE levels specific for 2xP (*y*-axes, OD values as box plots with indicated medians) measured before and at different days after sensitization (arrow S) and boosting (arrow B) (*x*-axes). Significant increases of IgE levels after boosting are indicated (*p < 0.05).

**FIGURE 7.**
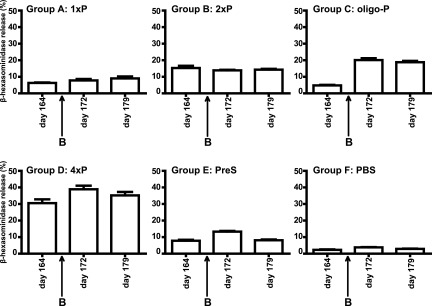
Induction of basophil degranulation with sera obtained from different groups of mice on days 164, 172, and 179. The time point of boosting (B) is indicated by an arrow. RBL cells were loaded with pooled serum IgE from the respective groups of mice (groups A–F) and challenged with a peptide–OVA conjugate. The mean β-hexosaminidase releases ± SD (*y*-axes) are shown for each group of mice on different days (*x*-axes) as percentages of total releases.

**FIGURE 8.**
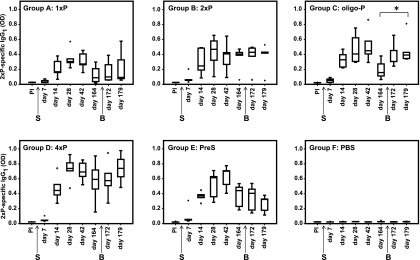
Peptide-specific IgG_1_ Ab levels over time in groups of mice (groups A–F). Shown are IgG_1_ levels specific for 2xP (*y*-axes, OD values as box plots with indicated medians) measured before and at different days after sensitization (arrow S) and boosting (arrow B) (*x*-axes). Significant increases of IgG_1_ levels after boost are indicated (*p < 0.05).

To investigate the boost of secondary IgE Ab responses, groups of mice (A–E, Fig. 2) were sensitized to 4xP by one s.c. immunization. The development of peptide-specific IgE levels was analyzed by ELISA and RBL assay for several months as indicated in Figs. 6 and 7. Single immunization of mice with 4xP led to the development of a primary IgE Ab response, which was detectable already on day 7, increased until day 14, and gradually started to decline from day 28 (Fig. 6, groups A–E). PBS did not induce peptide-specific IgE Ab response (Fig. 6, group F). As we sought to compare the secondary IgE Ab response to secondary IgG_1_ responses, the groups of mice were boosted on day 165, when we also observed a decline of peptide P–specific IgG_1_ Ab levels (Fig. 8). We studied which molecule among the various immunogens (Fig. 2) could boost peptide P–specific IgE Ab responses induced by 4xP immunization in groups A–E. Peptide-specific IgE Ab responses were significantly boosted only by oligo-P (*p* = 0.04) and 4xP (*p* = 0.04) (Fig. 6, groups C and D, Supplemental Fig. 2) whereas 1xP, 2xP, and the carrier alone (PreS) and PBS did not significantly boost peptide-specific IgE Ab responses (Fig. 6, groups A, B, E, and F, Supplemental Fig. 2). As the KLH conjugate oligo-P did not contain the carrier molecule used for sensitization (PreS), it did not receive help by PreS-specific T cells (Fig. 6, group C). No KLH-specific responses were detected in this mouse group, also excluding the help of KLH-specific T cells (data not shown).

Next, we investigated whether changes of peptide-specific IgE from day 164 to day 172 would have effects on basophil degranulation. For this purpose, pooled serum samples obtained at days 164, 172, and 179 from groups A–F were loaded on RBL-2H3 cells and degranulation was induced by a peptide–OVA conjugate, in which the peptide (P) was coupled to a carrier (OVA) with which the mice had not been in contact. The strongest increase of degranulation with the peptide–OVA conjugate was observed when basophils were loaded with sera from oligo-P–boosted mice from group C (i.e., 4.8% on day 164 to 20.1% on day 172) (Fig. 7, Supplemental Fig. 3), whereas no relevant alterations were noted for the other groups (group A, 1xP boosted, 6.3% on day 164 to 7.8% on day 172; group 2, 2xP boosted, 15.3% on day 164 to 13.9% on day 172; group D, 4xP boosted, 30.5% on day 164 to 38.9% on day 172; group E, PreS, 7.8% on day 164 to 13.3% on day 172) (Fig. 7). For the PBS group F, a background release of β-hexosaminidase of 3% was found for each of the three time points (Fig. 7).

### Peptide-specific IgG_1_ Ab responses are also boosted by oligo-P

We also studied the kinetics of IgG_1_ Abs in the same groups of mice that had received a single immunization with 4xP (Fig. 8). Interestingly, peptide-specific IgG_1_ responses started ∼1 wk later as compared with peptide-specific IgE responses (i.e., at day 14 after immunization) (Figs. 6, 8). Similarly, as observed for IgE responses (Fig. 6), oligo-P was able to significantly boost the peptide-specific IgG_1_ Ab levels (*p* < 0.05) (Fig. 8, group C), whereas monomeric peptide (1xP), dimeric peptide (2xP), and PreS did not induce boosts of peptide-specific IgG_1_ responses (Fig. 8). Interestingly, the boost of peptide-specific IgG_1_ levels by oligo-P occurred 1 wk later as the boost of peptide-specific IgE levels (Figs. 6, 8). Mice injected only with PBS did not produce any peptide-specific IgG_1_ responses, and there was also no boost of peptide-specific IgG_1_ in these mice (Fig. 8, group F). Boosting with 4xP also induced an increase of peptide-specific IgG_1_ Ab levels, but this did not reach statistical significance (Fig. 8, group D).

These results illustrate that oligo-P, which contained a different carrier molecule than that used for sensitization and therefore did not receive T cell help by carrier-specific T cells, can boost peptide-specific IgE and IgG_1_ Ab responses.

### T cell help for the 4xP-induced peptide-specific IgE and IgG Ab responses stems from the carrier protein PreS

**FIGURE 9.**
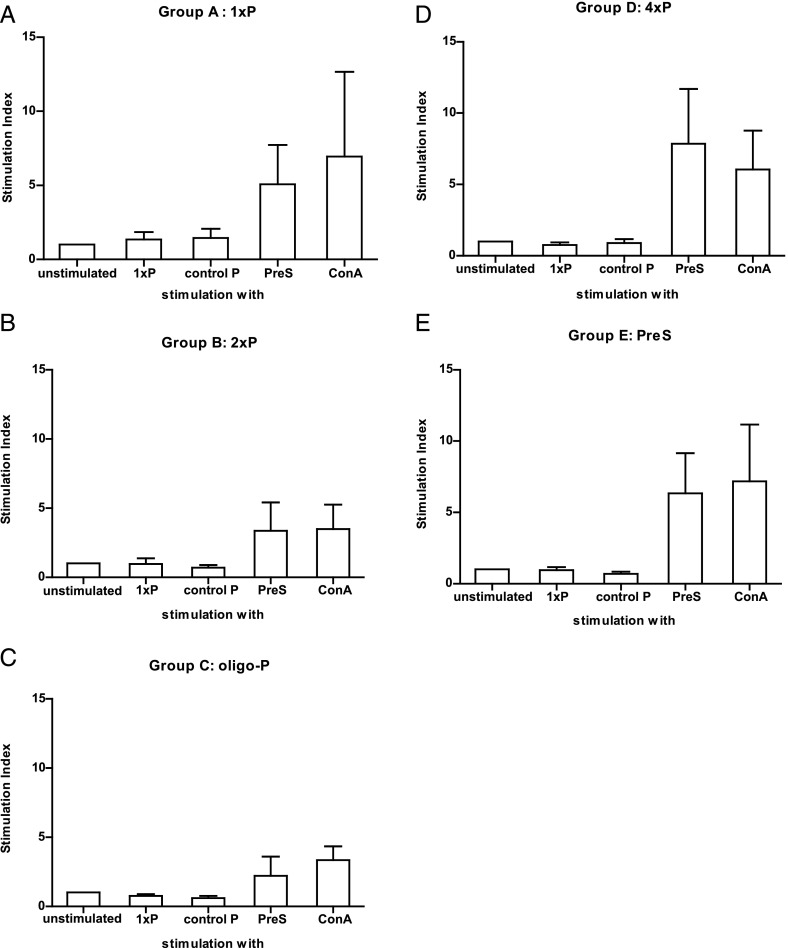
Peptide- and carrier-specific T cell responses in the sensitized and boosted mice. (**A**–**E**) Proliferations of mouse splenocytes of individual mice from groups A–E in response to 1xP, an unrelated control peptide from the major birch pollen allergen Bet v 1 (control P), PreS, Con A, or in the absence of stimuli (unstimulated) (*x*-axes) are shown as mean SIs ± SD (*y*-axes) on day 185.

Next we investigated the specificity of T cell help involved in the induction and boosting of peptide-specific IgE and IgG_1_ Ab responses. Spleen cells were isolated from the groups of mice that were immunized with 4xP and then boosted with the different Ags on day 185 (Fig. 2, groups A–E). Neither in response to peptide 1xP nor to an immunologically unrelated peptide from the major birch pollen allergen Bet v 1 (control P) was any relevant proliferation in spleen cells detectable in the tested mouse groups (Fig. 9, Supplemental Fig. 4). Only the carrier protein, PreS, which was part of the immunogen 4xP that was used for sensitization, induced a proliferation in splenocytes from mice of groups A–E (range, group C, oligo-P boosted, SI = 2.2 to group D, 4xP boosted, SI = 7.8; mean, SI = 3.72) (Fig. 9).

## Discussion

In this study we used one of the most important respiratory allergens, the major timothy grass pollen allergen Phl p 1, to investigate the contribution of allergen-derived B cell epitopes versus T cell epitopes on boosts of the secondary IgE response in a murine model. In fact, reducing the secondary IgE production in allergic patients is a long-sought goal that has been pursued as a possible therapeutic avenue with pharmacotherapy (23), anti-cytokine therapy (33), and IgE targeting approaches (34), but so far only SIT has been shown to have some pronounced effects on allergen-specific IgE production (17–19, 35).

In our model, allergic IgE sensitization was induced in mice with a fusion protein containing a peptide from the major IgE epitope–containing region of Phl p 1 linked to an allergen-unrelated carrier protein, the PreS protein from hepatitis B. Importantly, the fusion protein used for sensitization did not contain allergen-specific T cell epitopes. In fact, the major T cell epitope of Phl p 1 in BALB/c mice was mapped to the position 127–141 (27) of the allergen and accordingly we could not detect T cell responses toward Phl p 1 in the sensitized mice. T cell responses were directed exclusively toward the PreS carrier protein. In fact, only carrier-specific but no peptide-specific CD4^+^ T cells could be detected by FACS analysis and in proliferation assays.

Thus, immunization of mice with 4xP allowed the induction of a peptide-specific and thus allergen-specific IgE and IgG_1_ Ab response without detectable priming of allergen-specific T cells, whereas T cell help was provided by the carrier molecule PreS. The kinetics of the peptide-specific IgE and IgG_1_ responses in the sensitized mice were different. Peptide-specific IgE appeared earlier than peptide-specific IgG_1_ and declined already after 4 wk (day 28), whereas peptide-specific IgG_1_ levels remained high for a longer period (after day 42). The earlier rise of IgE might be explained by a direct class switch to IgE, which was hypothesized earlier (32, 36), whereas the longer increase of IgG might be explained by the longer half-life of IgG_1_ as compared with IgE (37) and/or due to differences in the IgE- and IgG-producing plasma cell populations. In fact, it has been reported that IgE-producing plasma cell populations are less long-lived (38, 39) than are IgG-producing plasma cells (40). We then used either the B cell epitope in a momoneric, dimeric, or oligomeric form (1xP, 2xP, oligo-P), the orginal immunogen (4xP) containing both T and B cell epitopes, or only the T cell epitope–containing part (i.e., PreS) for boosting without any adjuvant. Monomeric peptide (1xP) did not boost peptide-specific IgE Ab levels, which we attribute to its strictly monomeric nature shown by the biochemical analysis and hence by its inability to cross-link FcεRI-bound peptide-specific IgE on basophils. It therefore will also not be able to cross-link a BCR. Interestingly, also 2xP did not induce a significant boost of peptide-specific IgE responses, although this molecule induced effector cell activation and should in principle be able to cross-link the BCR on peptide-specific memory B cells. However, it is possible that a cross-linking of two B cell receptors is too weak of a signal and/or that the peptide preparation did not contain enough dimeric peptide. Alternatively, one may consider that the expression of membrane IgE on memory cells is lower than membrane IgG on memory B cells in mice (41). However, 2xP also did not induce a significant boost of peptide-specific IgG_1_ responses. Administration of the carrier molecule PreS containing only T cell epitopes to 4xP-sensitized mice had no effect on peptide-specific Ab responses. This is in accordance with results from a clinical study in birch pollen allergic patients, demonstrating the importance of intact B cell epitopes for boosting allergen-specific IgE (42). Only oligo-P and 4xP induced significant increases of peptide-specific IgE and IgG_1_ Ab responses. Because oligo-P did not induce any IgE responses against the carrier used in this molecule (i.e., KLH) (data not shown), we think that the increase of peptide-specific IgE response is not due to an induction of another “primary” IgE response but results from a direct stimulation of B memory cells by the oligomeric construct via cross-linking of the BCR. There are several possibilities for cross-linking of BCRs by allergens. First, it is possible that allergens contain repetitive IgE epitopes (43). Second, certain allergens tend to form aggregates and thus may offer multiple IgE epitopes (44, 45). Third, it is possible that in the context of a polyclonal Ab response against allergens, Abs may supercross-link an allergen when bound to the BCR, a mechanism that has been reported to also augment mast cell degranulation (46, 47). T cell–independent, direct boost of IgE production via cross-linking of the BCR would explain several observations pointing toward T cell independence of boosts of secondary IgE production such as the inability to control boosts of IgE production by suppression of T cell responses with T cell–targeting drugs (22, 23) or the occurrence of boosts of IgE production in patients with insufficient numbers of T cells (21). So far a reduction of the boosts of allergen-induced IgE production has only been observed under allergen-specific immunotherapy, which may be explained by the occupation of IgE epitopes on allergens by therapy-induced IgG, which would render them incapable of cross-linking the BCR on IgE memory cells (17–19). However, besides the induction of allergen-specific–blocking IgG Abs in allergic patients during immunotherapy treatment, other complex regulatory mechanisms such as the induction of regulatory T cell populations, cytokine effects, or the influence of certain APCs may also play a role.

We think that our findings are also important for the design of new forms of SIT, because it may be considered to use recombinant allergen derivatives that primarily induce allergen-specific IgG responses without activating allergen-specific T cell epitopes (4, 47–50) for induction of IgE to downregulate allergen-specific IgE responses.

## Supplementary Material

Data Supplement
