# Supplementary material for: A B Cell Epitope Peptide Derived from the Major Grass Pollen Allergen Phl p 1 Boosts Allergen-Specific Secondary Antibody Responses without Allergen-Specific T Cell Help
Source: J Immunol. 2017 Jan 16;198(4):1685–95. doi: 10.4049/jimmunol.1501741 (PMC5292585; doi:10.4049/jimmunol.1501741)
Supplement: Data Supplement [file JI_1501741.zip › JI_1501741_Supplemental_Figures_1.pdf]

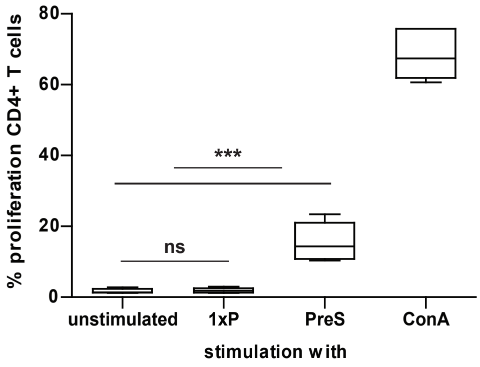

Figure S1. Demonstration of the carrier-specificity of the T cell response in 4xP sensitized mice. BALB/c mice were sensitized and boosted 3 times with 4xP. Proliferation of CD4+ cells (y-axis) in VPD-450-labeled splenocyte cultures of each mouse was analyzed by FACS after *in vitro* stimulation with 1xP, PreS, Con A or in unstimulated cells (x-axis) and is depicted as box blots. Box and whiskers demonstrate min to max and median with standard deviation. Differences between groups were analyzed with one way ANOVA and Tukey´s multiple comparisons test in GraphPad Prism 6. Data are pooled data of 2 independent experiments (n=5).

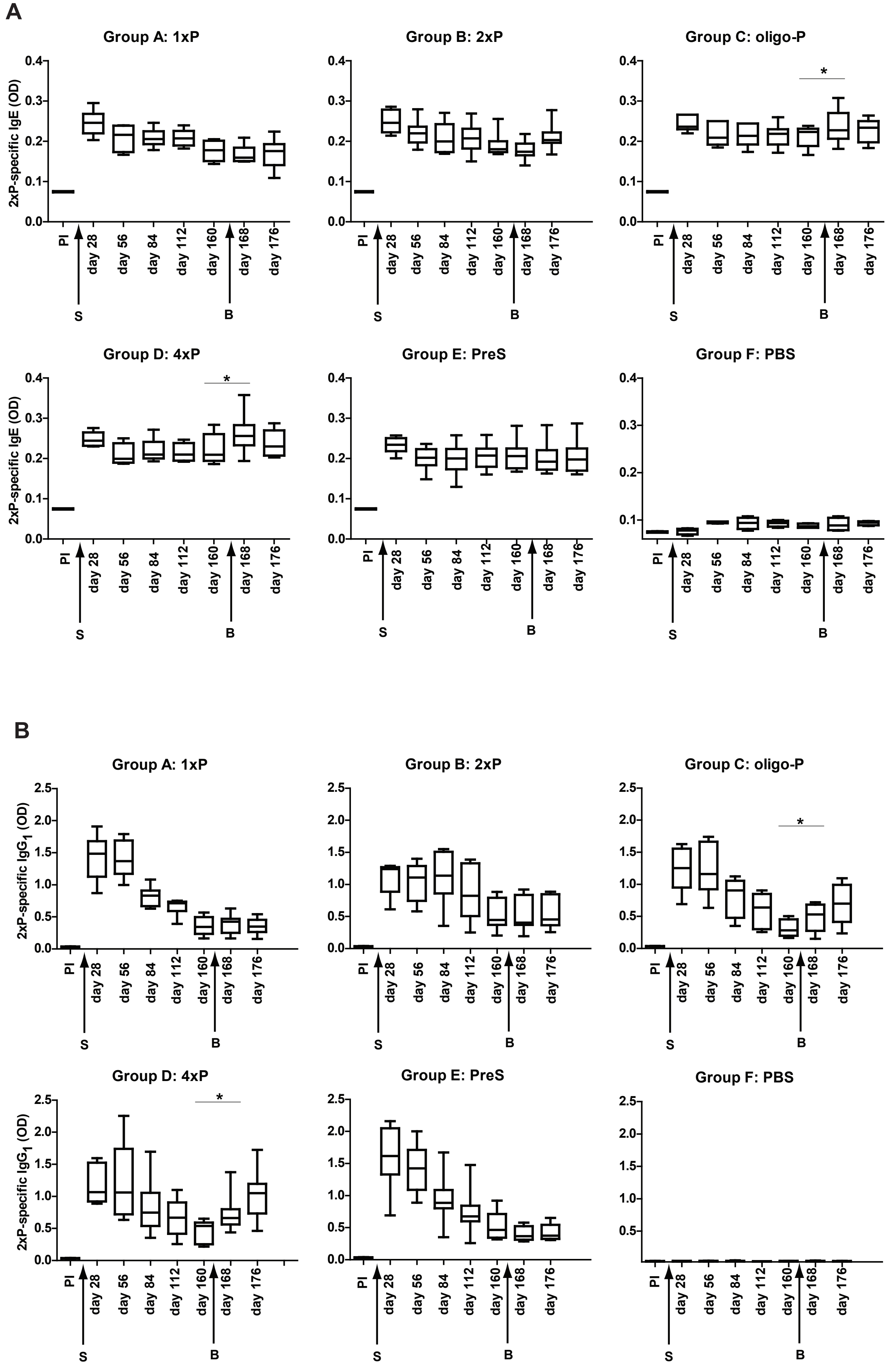

Figure S2. Peptide-specific IgE and IgG_1 antibody levels over time in mouse groups A-F (n=8). Results derive from a second independent experiment and replicate the data shown in Figures 6 and 8. Shown are IgE (A) and IgG_1 (B) levels (y-axes: OD values as box plots with indicated medians) measured before and at different days after sensitization (arrow S) and boosting (arrow B) (x-axes). Significant increases of IgE levels after boosting are indicated by asterisks (* $p<0.05$).

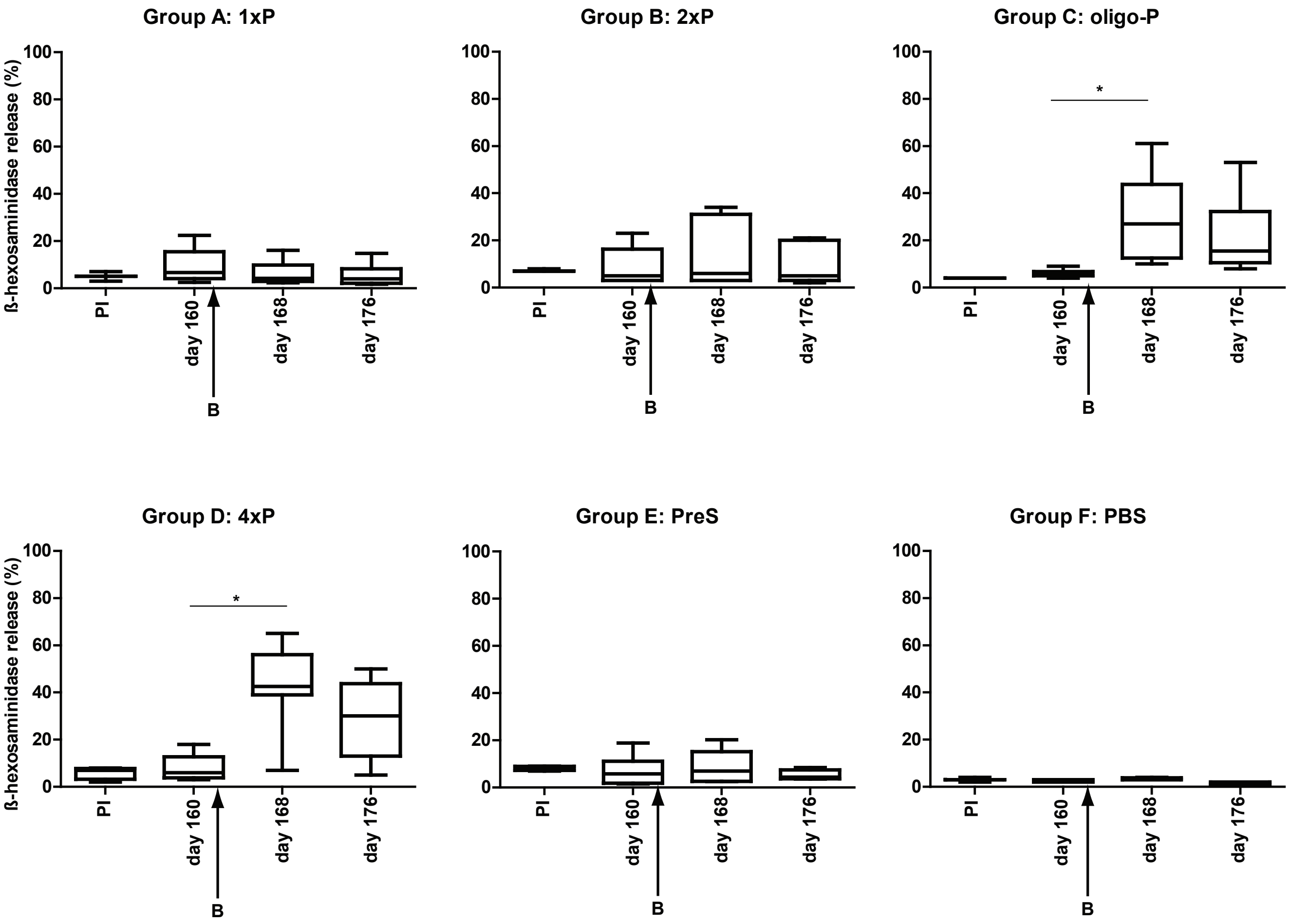

Figure S3. Induction of basophil degranulation with sera obtained from mouse groups A-F (n=8) before sensitization (PI), on various days after sensitization and boosting (arrow B). Results come from a second independent experiment and replicate the data shown in Figure 7. RBL cells were loaded with individual mouse sera from the respective groups of mice and challenged with a peptide-ovalbumin conjugate. The mean β-hexosaminidase releases (y-axes: box plots with indicated medians) are shown for each group of mice on different days (x-axes) as percentages of total releases. Significant increases of β-hexosaminidase releases after boosting are indicated by asterisks (* $p<0.05$).

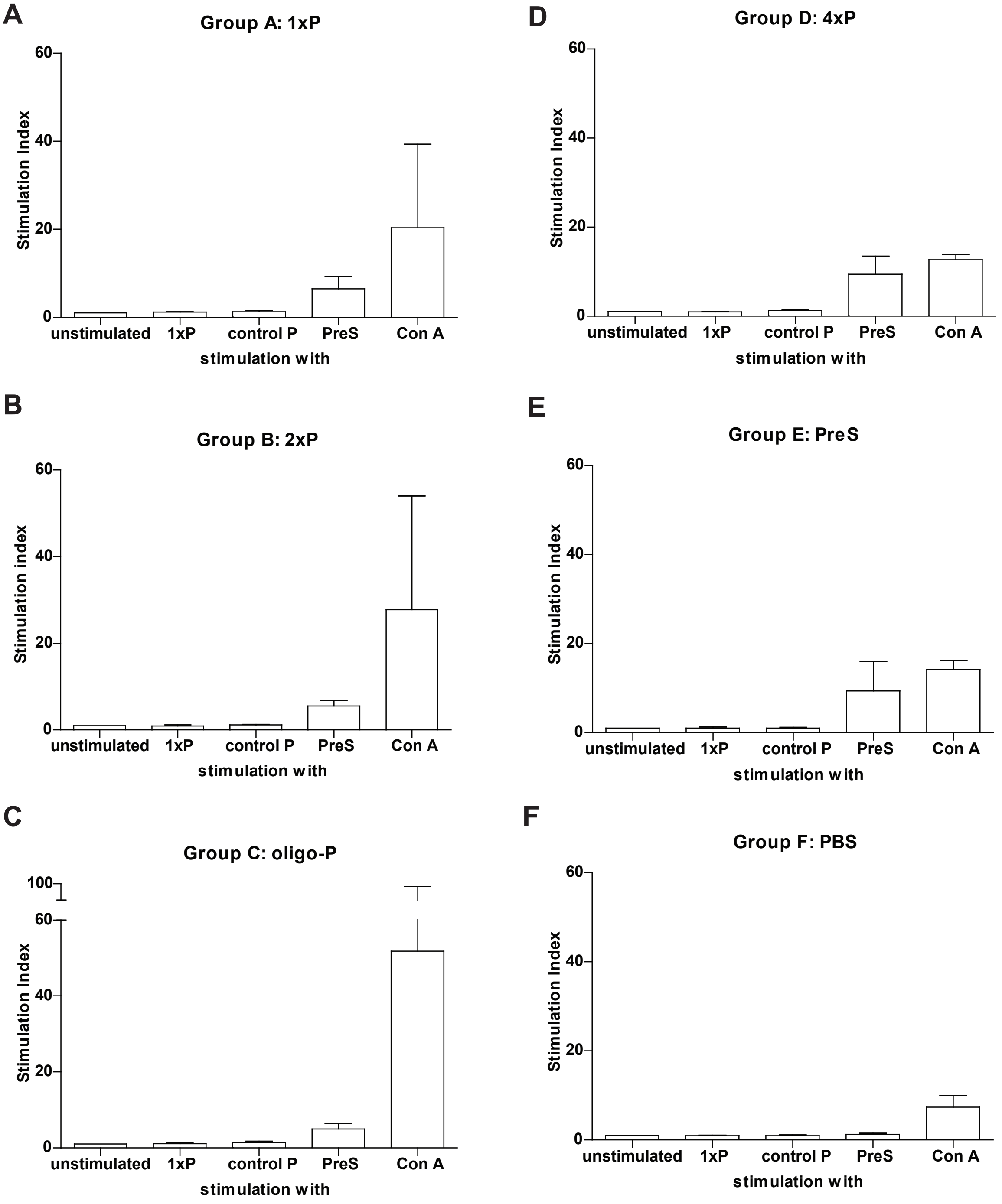

Figure S4. Peptide and carrier-specific T cell responses in the sensitized and boosted mice. Results derive from a second independent experiment and replicate the data shown in Figure 9. Shown are the proliferations in response to 1xP, an unrelated peptide from the major birch pollen allergen Bet v 1 (control P), PreS, ConA, and of unstimulated cells (x-axes) as mean stimulation indices (SIs) +/- SDs (y-axes) obtained for splenocytes of individual mice from groups A-F on day 185.
